# Capture Myopathy and Stress Cardiomyopathy in a Live-Stranded Risso’s Dolphin (*Grampus griseus*) in Rehabilitation

**DOI:** 10.3390/ani10020220

**Published:** 2020-01-29

**Authors:** Nakita Câmara, Eva Sierra, Antonio Fernández, Manuel Arbelo, Yara Bernaldo de Quirós, Marina Arregui, Francesco Consoli, Pedro Herráez

**Affiliations:** 1 Veterinary Histology and Pathology, Institute of Animal Health and Food Safety (IUSA), Veterinary School, University of Las Palmas de Gran Canaria, Arucas, 35416 Las Palmas de Gran Canaria, Spain; kita_camara@hotmail.com (N.C.); antonio.fernandez@ulpgc.es (A.F.); manuel.arbelo@ulpgc.es (M.A.); yara.bernaldo@ulpgc.es (Y.B.d.Q.); marina.arregui@ulpgc.es (M.A.); francesco.consoli@studio.unibo.it (F.C.); pedro.herraez@ulpgc.es (P.H.); 2 Department of Neuroscience, Imaging and Clinical Sciences, University G. D’Annunzio, 66100 Chieti, Italy

**Keywords:** animal conservation, animal welfare, cetaceans, biochemistry, histopathology, immunohistochemistry

## Abstract

**Simple Summary:**

Free-living cetaceans are threatened, daily, by a wide variety of stressful situations. An example is provided by live-stranding, in which a cetacean is alive on the beach or in shallow water, and unable to free itself and resume its normal activity. This is the first case of capture myopathy and stress cardiomyopathy in a live-stranded juvenile male Risso’s dolphin (*Grampus griseus*) with subsequent rehabilitation attempted. Valuable use of blood samples, and finally necropsy assessments, advances our understanding about the pathology common in live-stranded cetaceans.

**Abstract:**

Capture myopathy (CM) is described in wild animals as a metabolic syndrome resulting from the extreme stress suffered during and after capture, handling, restraint, and transport. Although CM has been characterized in many species of cetaceans, descriptions of cardiac injury—an important component of this syndrome, and, according to previous authors, comparable to the existing human pathology so-called stress cardiomyopathy (SCMP)—are still rare. Therefore, the main aim of this report is to illustrate, for the first time, the biochemical analysis, and gross, histopathological, histochemical and immunohistochemical features of CM, and more specifically of the SCMP involved in this syndrome, caused by the live-stranding and consequent rehabilitation attempt, for a certain period of time, in a juvenile male Risso’s dolphin (*Grampus griseus*). The animal presented elevated values of creatine kinase, cardiac troponin I and blood urea nitrogen, with some variations during the rehabilitation period. Histologically, we detected vascular changes and acute degenerative lesions analogous to the ones observed in humans with SCMP. We consider this study to be an important contribution to the study of cetaceans since it could help in decision-making and treatment procedures during live-strandings and improve conservation efforts by reducing the mortality of these animals.

## 1. Introduction

Stress is present in daily life, and all life forms have evolved mechanisms to cope with stressful situations [1]. It is commonly defined as the adaptive changes (or stress responses) caused when a person or animal perceives a threat to their homeostasis [2,3].

In human pathology, despite still under-recognized and often misdiagnosed, an unique reversible cardiac syndrome, triggered by a stressful event, has globally been reported and described as stress cardiomyopathy (SCMP) [4,5].

The clinical presentation is similar to the acute myocardial infarction. Diagnostic criteria for SCMP has been established by the Heart Failure Association (electrocardiography, echocardiography, cardiac catheterizations, and biochemical analysis). Regarding the biomarkers, cardiac troponin I (cTnI) and/or creatine kinase (CK) have small, rapid increases to above normal levels [4,5,6,7].

Histological findings are consistent with ischemia-reperfusion injury, such as vascular changes (i.e., congestion, hemorrhages, interstitial edema) and acute necrotic degenerative lesions (i.e., contraction band necrosis, wavy fibers, hypereosinophilia and cytoplasmic vacuolization) [4,5,8]. 

Cetaceans are exposed daily to a wide variety of stressful situations (i.e., live-stranding, ship collisions, bycatch) that influence their well-being. Efforts have been made to reduce the impact of these situations, but whales and dolphins continue to be threatened [9,10,11,12,13,14,15]. Live-stranding is an unnatural and distressful situation, where the cetacean is alive on the beach or in shallow water, being unable to resume normal activity [16,17,18]. Independently of the animal’s previous health, this implies an anomalous and extreme situation for an organism that is not adapted to terrestrial environmental conditions. Therefore, it is considered life-threatening as it may cause death or seriously aggravate a previous disease [9,10,11,12,14,16,18,19].

The acute and stressful deaths of live-stranded cetaceans might be attributed to the “stress response syndrome” or “alarm reaction” which are thought to be comparable to those described in capture myopathy (CM), in which the cardiac damage, due to the extreme stress, seems to have an important role. According to some authors, cetaceans are especially predisposed to develop SCMP, similarly to the SCMP in humans. It is the most devastating form of acute stress described in several animals that may occur during and after the capture, handling, restraint, and transport [9,10,14]. Although the pathological findings vary amongst individuals, the biochemical changes are consistent with elevated serum muscle enzyme activities, specifically CK, and elevated blood urea nitrogen (BUN). There are no biochemical studies on *Odontoceti* in which specific cardiac markers have been analyzed. Histopathological changes consisting of ischemia-reperfusion injuries may be observed. These changes result in local-to-generalized vasospasms and vasodilation (as seen in human SCMP), as well as in direct traumatic injury to muscles (rhabdomyolysis), with acute renal failure associated with myoglobinuric nephrosis secondary to muscle damage [9,10,11,12,15,20,21].

The aim of the present report is to illustrate, for the first time, the biochemical analysis, gross, histopathological, histochemical and immunohistochemical features of the CM, and more specifically of the SCMP involved in CM, caused by the live-stranding and consequent rehabilitation attempt in a juvenile male Risso’s dolphin (*Grampus griseus*).

## 2. Materials and Methods

This study is of a juvenile male Risso’s dolphin (*Grampus griseus*) which was stranded alive on the coast of Gran Canaria (Canary Islands, Spain), on April 26, 2019 (notification at 8:30 a.m.). After receiving specialized first aid (i.e., help maintaining correct body posture and wetness of skin, evaluating breathing and heart rate) at the location, the animal was transferred to the Wildlife Recovery Center (Gran Canaria), where it was monitored 24 h a day. From day 0 (26 April 2019) to day 5 (1 May 12019), several diagnostic tests (i.e., biochemical and hematological analysis, gastric probing and ultrasound evaluation) were performed to establish the most appropriate treatment. Around 10 p.m. on 29 April 2019 (day 3), the animal began to have muscle spasms. The next day, a lateral body curvature began to develop at the caudal peduncle, with increasing muscle tremors. Due to the worsening prognosis the animal was euthanized on 1 May 2019 (day 5). The therapeutic treatment and the euthanasia protocol applied in this animal are described in the Supplementary Materials Tables S1 and S2, respectively.

### 2.1. Evidence of Ethical Approval

Permission for the management of stranded cetaceans was issued by the environmental department of the Canary Islands’ Government and the Spanish Ministry of Environment. 

### 2.2. Biochemical Analysis

Samples of whole blood were collected from the tail flukes, into a gel tube (without anticoagulant), were allowed to coagulate, and then centrifuged twice, each time at 3500 rpm for 5 min to obtain the serum for analysis. The first blood sample was collected 4 h after the warning (approximately midday), the remaining samples every 24 h during rehabilitation, and also post-mortem (1:30 h after euthanasia). The biomarkers for acute skeletal and heart muscle damage (CK and cTnI) and the kidney function (BUN and creatinine) were analyzed. 

### 2.3. Gross, Histological, Histochemical and Immuznohistochemical Analysis

Prior to the necropsy, and after the euthanasia, an entire body scan was carried out, using helical computer tomography (CT) (Toshiba Astellion 16) setting the protocol on 120kV, 300 mA and a reconstruction slice thickness/slice interval of 1/0.80 mm. All the DICOM files obtained were processed with the Horos software. Thereafter, a standard protocol full necropsy was performed, where representative tissue samples were collected and fixed in 10% formalin for approximately 48 h and processed using standard protocol [16,22]. Due to the potential presence of CM and SCMP, the skeletal (*longissimus dorsi* and *rectus abdominis*) and heart muscles (both atria and ventricles), atrioventricular valves (bicuspid or mitral and tricuspid), semilunar valves (sigmoid aortic and sigmoid pulmonary with the corresponding arteries), and kidneys were fully studied. Tissue sections (4- and 5-µm-thick) were used for hematoxylin and eosin (HE) and Masson’s trichrome technique, respectively, while 3-µm-thick layers were immunolabeled with anti-myoglobin, anti-fibrinogen, anti-cardiac troponin I, and anti-cardiac troponin C primary antibodies. The followed protocol was presented in a previous publication, and it can be accessed in Supplementary Materials Table S3 [15].

## 3. Results

### 3.1. Biochemical Results

**Figure 1:**
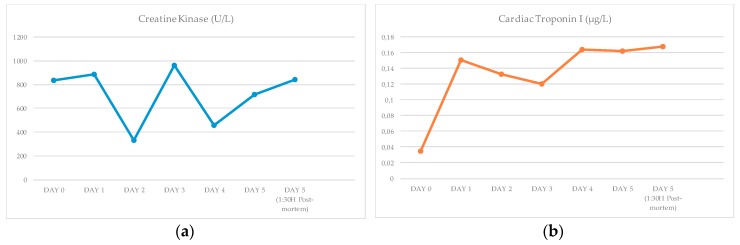

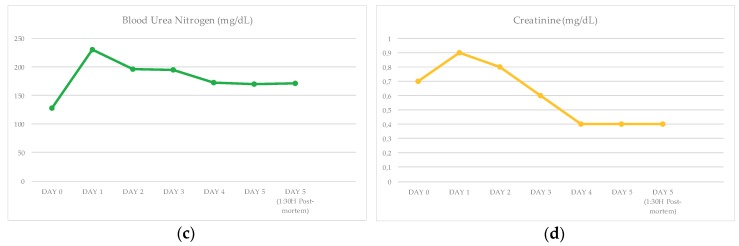
Biochemical analysis during rehabilitation and after euthanasia: (**a**) Creatine kinase (CK); (**b**) Cardiac troponin I (cTnI); (**c**) Blood urea nitrogen (BUN); (**d**) Creatinine.

The different measurements obtained during the rehabilitation time and after euthanasia, are presented in Figure 1 and in the Supplementary Materials Table S4. 

It was possible to observe that every single result obtained for CK was high. It is important to remark the new peak of the value on day 3. This animal presented one value within the normal range on day 0 for cTnI, being the rest of the measurements elevated. As noticed in the CK, the cTnI also presented a peak on day 4 and 5–1:30 h after euthanasia. The animal demonstrated higher BUN values and lower creatinine level than normal values.

### 3.2. Gross Results

**Figure 2:**
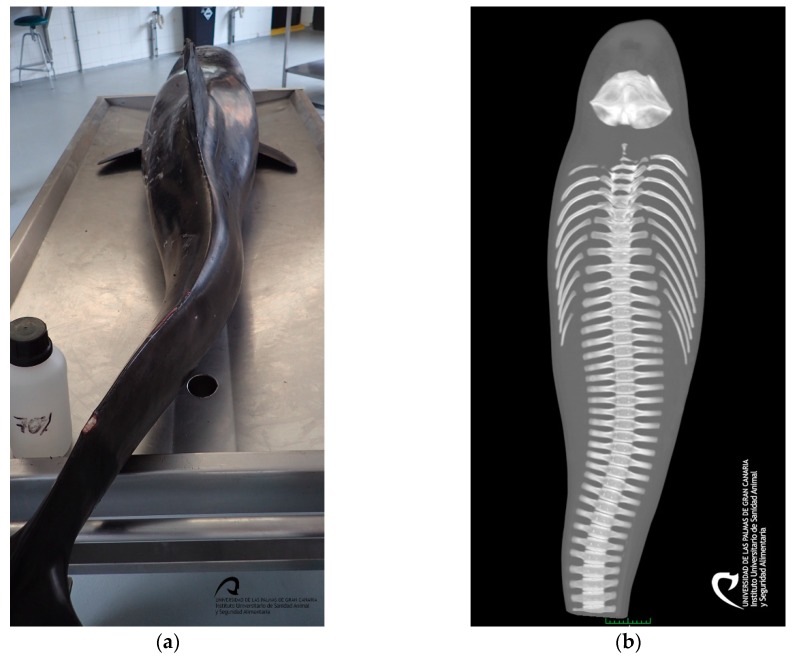

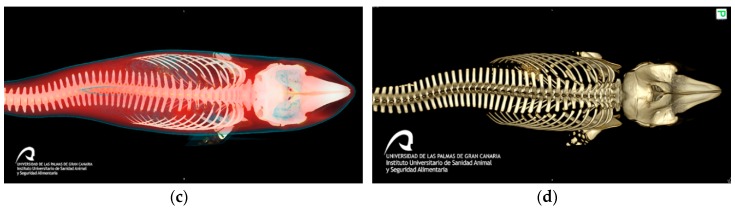
Animal caudal peduncle scoliosis: (**a**) Caudal view on the necropsy table; (**b**) Dorsal plane of the entire body, with computer tomography (CT); (**c**) Dorsal view of the entire body, using CT, bones in white, muscle in red, and skin in blue; and (**d**) Dorsal view of the skeleton, with the CT.

The 105 kg juvenile male, measured 205.5 cm, had a very poor body condition, and displayed several lacerations, distributed in a multifocal manner, on the rostrum, dorsal and pectoral fins, tail flukes and ventral part of the body, attributed to live-stranding. External and internal signs of a spinal lateral curvature (S-shape) were identified at the level of the caudal peduncle (Figure 2).

### 3.3. Histopathological Results

**Figure 3:**
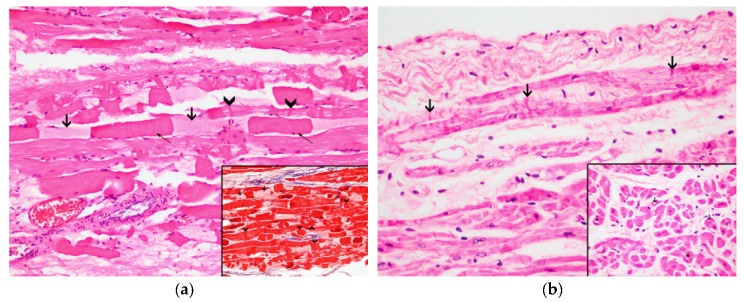
Histochemical techniques: (**a**) Contraction band necrosis (arrow heads), segmentary fibrilar degeneration (thick arrows) with hyalinized eosinophilic sarcoplasm and hypercontraction (thin arrows); 20x, HE. Inset: Detail of the myocytes with contraction band necrosis (arrow heads) and segmentary fibrilar degeneration (thick arrows) with hyalinized eosinophilic sarcoplasm and hypercontraction (thin arrows); 20×, Masson’s trichrome technique; (**b**) Contraction band necrosis (arrows) in the cardiomyocytes; 40×. Inset: Detail of the intracytoplasmic vacuoles (arrows) of the cardiomyocytes; 60×, HE.

On histopathologic and histochemical examinations, the skeletal and heart muscles presented injuries consistent with acute degenerative lesions. The *longissimus dorsi* and the *rectus abdominis* demonstrated a multifocal, severe contraction band necrosis and myonecrosis consisting of segmentary fibrilar degeneration with hyalinized eosinophilic sarcoplasm and hypercontraction (Figure 3a). The atria and ventricles displayed a multifocal, moderate degree of contraction band necrosis (Figure 3b), wavy fibers, hypereosinophilia, and cytoplasmic vacuolization (Figure 3b, inset) with pyknotic nucleus. All of the above changes were more pronounced in the subepicardial and subendocardial regions. The different heart sections also exhibited a multifocal, mild degree of infiltration of mononuclear cells in the areas with fibrillary ruptures. 

The kidneys showed a multifocal, moderate congestion in the cortex and renal medulla. An amorphous and acidophilic substance was found inside the Bowman’s capsule and in the medullary renal tubules.

### 3.4. Immunohistochemical Results

**Figure 4:**
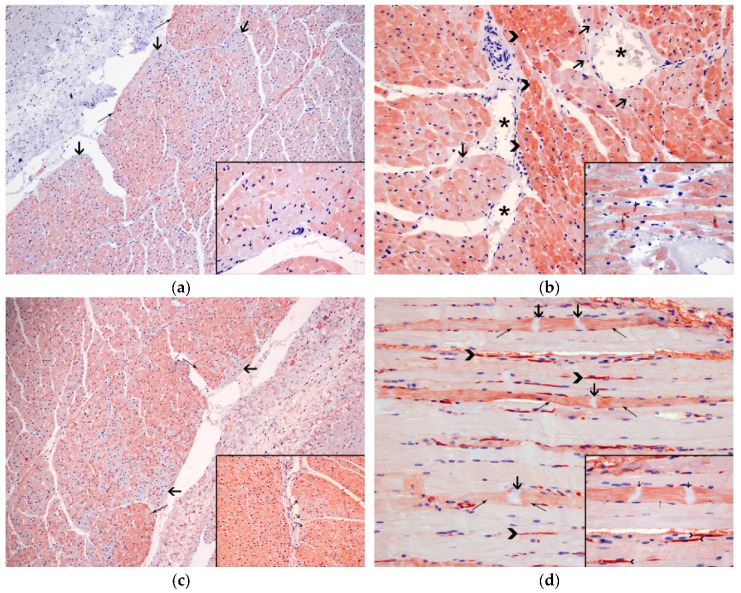
Immunohistochemical techniques: (**a**) Degenerated cardiomyocytes (thick arrows) present depletion of cardiac troponin I (cTnI), compared to the normal cardiomyocytes (arrow heads). 10×. Inset: Detail of the intrafibrillar depletion (thick arrows) of cTnI in the injured cardiomyocytes, in comparison with normal cardiomyocytes (thin arrows); 60×, anti-troponin I; (**b**) Necrotic cardiomyocytes (thick arrows), close to the blood vessels (*) demonstrate depletion of cardiac troponin C (cTnC), compared to the normal cardiomyocytes (arrow heads); 20×. Inset: Detail of intrafibrillar depletion of cTnC with intense immunolabelling in the contraction band necrosis (thick arrows); 60×, anti-troponin C; (**c**) Degenerated cardiomyocytes (thick arrows), show intrafibrillar depletion of myoglobin, compared to the normal cardiomyocytes (thin arrows); 10×. Inset: Detail of the intrafibrillar depletion (thick arrows) of myoglobin in the injured cardiomyocytes, in comparison with normal cardiomyocytes (thin arrows); 20×, anti-myoglobin; (**d**) Expression of fibrinogen, in the necrotic myocytes, mainly in area next to (thin arrows) the contraction band necrosis (thick arrows). Presence of fibrinogen in the blood vessels (arrow head); 20×. Inset: Detail of the immunolabelling of fibrinogen, in the injured myocytes, in the zone near to (thin arrows) the contraction band necrosis (thick arrows). Fibrinogen inside the blood vessels (arrow heads); 60× anti-fibrinogen.

The degenerated/necrotic muscular and heart cells showed homogenous, intrafibrillar depletion of cTnI (Figure 4a), cardiac troponin C (cTnC) (Figure 4b), and myoglobin (Figure 4c), and exhibited immunolabelling for fibrinogen (Figure 4d). The kidneys did not exhibit any displacement/presence of myoglobin.

## 4. Discussion

### 4.1. Discussion of the Biochemical Results

Clinico-pathological evaluation was challenging, not only because normal and/or pathological biochemical values are rarely reported for cetaceans, and in this case, particularly regarding Risso’s dolphins but also due to the non-existing normal range for specific cardiac markers, such as cTnI, in the cetacean scientific database. Our biochemical analysis was compared with different mammals in published papers [23,24,25,26].

The most relevant biochemical changes described in CM in birds (i.e., wild turkeys, mallards), terrestrial (i.e., hoofed mammals), and marine wild mammals (i.e., sea otters, seals), are the CK and BUN elevation [9,10,11,12,19,21,27]. For SCMP in humans, the more common laboratories abnormalities are consistent with a small, rapid rise to above the normal levels of cTnI and/or CK [6,7]. 

After an injury to the muscle (both skeletal and cardiac), the serum level of CK begins to rise in 4–9 h, peaks at 24 h, and returns to baseline 48–72 h, unless a new injury or permanent damage occurs [28]. Every single result obtained is higher than the normal range published for this species (48 to 154 U/L) which is a useful indicator of both skeletal and cardiac muscle damage suffered by this animal during and after stranding, and also during the rehabilitation period [18,19,21,26,28]. Additionally, the highest value of this marker on day 3 indicates the occurrence of a new injury. 

Although CK is considered a sensitive marker of myocardial damage, it is present in the skeletal muscles in high concentrations. Due to its poor specificity to detect specific heart damage, troponins have been adopted as the new gold standard for necrosis, more specifically cTnI, since it is detectable in very low amounts (0.01 µg/L) in the blood of healthy individuals [28,29]. Thus, significant elevations (≥ 0.1 µg/L) most likely reflect myocardial necrosis [28]. It is considered as cardiospecific, therefore it is used to detect heart pathologies (i.e., SCMP) [6,28,29]. Most of the injured cardiac myocytes release troponin, 3–9 h after ischemic damage, reaching its peak between 12–48 h and remains raised for up to 4–7 days, unless a new injury or permanent damage occurs [28,29]. 

At present, there are no published studies on *Odontoceti* that analyze specific cardiac markers to detect the resulting damage to the heart in these animals. Compared to the recommended values in humans (≤0.1 µg/L) and in dogs (≤0.03–0.07 µg/L), this animal presented one value within the normal range on day 0 (0.035 µg/L), being the rest of the measurements elevated [23,24,25]. All the results concur with the kinetics except on day 4 and 5 - 1:30 h after euthanasia, which indicates the occurrence of a new stress event, leading to a new damage. In this case, pre-renal azotemia was reasonably supported by higher BUN values than the existing reference values for this species (i.e., 36 to 69 mg/dL), associating this clinical finding with various possible causes, such as compartment syndrome, heart failure, and dehydration [19,26,30]. All measurements of creatinine were below the normal range for this species (1.4 to 2.8 mg/dL) probably related to the poor body condition [26,30]. 

### 4.2. Discussion of the Gross, Histopathological and Immunohistochemical Results

The chronology of events described in this case report is concurring with previous cases of live-stranded cetaceans, being the scoliosis a common problem in these animals, occurring during the rehabilitation process secondary to lack of swimming in a compromised animal [18,31]. 

The histopathological findings present in animals who suffered from CM consist of acute to subacute degeneration (rhabdomyolysis) and acute renal failure associated with myoglobinuric nephrosis secondary to muscle damage. The alterations related with the SCMP present in CM are associated with morphological alterations namely acute degenerative lesions (i.e., contraction band necrosis, wavy fibers, hypereosinophilia, and cytoplasm vacuolization), vascular changes (i.e., congestion, interstitial edema, and hemorrhages) and infiltration by inflammatory cells, based on the histological analysis of myocardial tissue [4,8,9,10,11,15,19,21,27]. Baring this in mind, the skeletal muscle and kidney samples of this animal presented lesions which illustrated rhabdomyolysis and acute renal failure. In relation to the heart lesions it is possible to conclude that the animal was entering the subacute stage (1 to 3 days), described in the sequence of changes in an acute ischemic injury in the heart, since it is when lymphocytes start to appear [5]. Immediately after a vital trauma, skeletal and cardiac proteins begin to leak as a result of early cell membrane rupture, causing a quick decline in myoglobin, cTnI, and cTnC contents, along with the deposition of plasma proteins (fibrinogen) [9,10,15,32,33]. These changes were observed in our animal confirming the ante mortem lesions. 

## 5. Conclusions

We consider this article to be an important contribution to clinical biochemistry in cetaceans. Valuable use of blood samples and finally necropsy assessment advances our understanding about the pathology in live-stranded cetaceans. 

## Supplementary Materials

The following are available online at https://www.mdpi.com/2076-2615/10/2/220/s1, Table S1: Summary of the therapeutic treatment. Table S2: Summary of the euthanasia protocol. Table S3: Summary of the immunohistochemical methodology. Table S4: Comparison between the biochemical analysis during rehabilitation (ante-mortem) and after euthanasia (post-mortem) with the normal baseline values.
