# Supplementary material for: Capture Myopathy and Stress Cardiomyopathy in a Live-Stranded Risso’s Dolphin (Grampus griseus) in Rehabilitation
Source: Animals (Basel). 2020 Jan 29;10(2):220. doi: 10.3390/ani10020220 (PMC7070958; doi:10.3390/ani10020220)
Supplement: Supplementary file 1 [file animals-10-00220-s001.pdf]

Table S1. Summary of the therapeutic treatment.

Medicine	Active Principle	Dosage [1]	Number of Dosages	Via	Days
Dexametasona Kern Pharma	Dexamethasone	0.11 mg/kg	1	Intramuscular	Day 0 (26/04/2019)
Ganadexil Enrofloxacino	Enrofloxacin	5 mg/kg	1 (each day)	Intramuscular	Day 0 (26/04/2019)<br>Day 1 (27/04/2019)<br>Day 2 (28/04/2019)
				Oral (diluted at 50%)	Day 3 (29/04/2019)<br>Day 4 (30/04/2019)
Zipyran Plus	Praziquantel	10 mg/kg	1	Oral	Day 1 (27/04/2019)
Ketoisdin	Ketoconazole	5 mg/kg	1	Oral	Day 2 (28/04/2019)
Fluid therapy	Freshwater	2 liters	2	Oral	Day 2 (28/04/2019)
			1 (each day)		Day 3 (29/04/2019)<br>Day 4 (30/04/2019)

1. Gulland, F.M.D.; Dierauf, L.A.; Whitman, K.L. *CRC handbook of marine mammal medicine*; CRC Press, 2018; ISBN 1351384163.

Table S2. Summary of the euthanasia protocol.

Medicine	Active Principle	Dosage	Number of Dosages	Via	Days	Commentaries
Tiobarbital	Sodium Thiopental	5 grams	1	Intravenous	Day 5 (01/05/2019)	The decision to euthanize the animal was taken among several veterinarians who are experts in dealing with these types of animals, one of them being the veterinarian responsible for the Wildlife Recovery Center (Gran Canaria) and veterinarians of Loro Parque. The main reason, which lead to this decision, consisted in the development of the body curvature which could not be reversible seeing that there were no adequate facilities to maintain the animal, more specifically, a pool which allow the animal to have a proper swimming pattern and not having the access to the therapeutic treatment recommend to reverse scoliosis in cetaceans in rehabilitation.

Table S3. Summary of the immunohistochemical methodology used in this study.

Antigen Retrieval	Serum	Source	Dilution	Primary Antibody	Source	Host	Type	Dilution	Secondary Antibody	Source	Dilution
Citrate buffer[1]	Swine serum[3]	Dako[4]	10%[5]	Myoglobin[6]	Abcam[10]	Rabbit	Polyclonal	1 in 200[11]	Polyclonal Swine Anti-Rabbit Immunoglobulins[15]	Dako[4]	1 in 200[16]
Citrate buffer[1]	Swine serum[3]	Dako[4]	10%[5]	Fibrinogen[7]	Abcam[10]	Rabbit	Polyclonal	1 in 50[12]	Polyclonal Swine Anti-Rabbit Immunoglobulins[15]	Dako[4]	1 in 200[16]
Citrate buffer[2]	Swine serum[3]	Dako[4]	10%[5]	Troponin I[8]	Abcam[10]	Rabbit	Polyclonal	1 in 25[13]	Polyclonal Swine Anti-Rabbit Immunoglobulins[15]	Dako[4]	1 in 200[16]
Citrate buffer[2]	Swine serum[3]	Dako[4]	10%[5]	Troponin C[9]	Abcam[10]	Rabbit	Monoclonal	1 in 250[14]	Polyclonal Swine Anti-Rabbit Immunoglobulins[15]	Dako[4]	1 in 100[17]

[1] Citrate buffer, pH 6.0, 7 minutes at 96ºC. [2] Citrate buffer, pH 6.0, 20 minutes at 96ºC. [3] Dako Swine serum (Normal) (X090110-8). [4] Dako (Glostrup, Denmark). [5] Dilution of 10 µl of serum in 90 µl of PBS. The serum is incubated in a humidity chamber for half an hour. [6] Anti-Myoglobin antibody (ab187506). [7] Anti-Fibrinogen antibody (ab34269). [8] Anti-Cardiac Troponin I antibody (ab47003). [9] Anti-Cardiac Troponin C antibody (ab137130). [10] Abcam (Cambridge, United Kingdom). [11] Dilution of 1 µl of primary antibody in 199 µl of serum at 1% in PBS (dilution of 1 µl of serum in 99 µl of PBS). The primary antibody is incubated in a humidity chamber for at least 18 hours, inside the refrigerator. [12] Dilution of 1 µl of primary antibody in 49 µl of serum at 1% in PBS (dilution of 1 µl of serum in 99 µl of PBS). The primary antibody is incubated in a humidity chamber for at least 18 hours, inside the refrigerator. [13] Dilution of 1 µl of primary antibody in 24 µl of serum at 1% in PBS (dilution of 1 µl of serum in 99 µl of PBS). The primary antibody is incubated in a humidity chamber for at least 18 hours, inside the refrigerator. [14] Dilution of 1 µl of primary antibody in 249 µl of serum at 1% in PBS (dilution of 1 µl of serum in 99 µl of PBS). The primary antibody is incubated in a humidity chamber for at least 18 hours, inside the refrigerator. [15] Dako Polyclonal Swine Anti-Rabbit Immunoglobulins/Biotinylated (E035301-2). [16] Dilution of 1 µl of secondary antibody in 199 µl of serum at 1% in PBS (dilution of 1 µl of serum in 99 µl of PBS). The secondary antibody is incubated in a humidity chamber for half an hour. [17] Dilution of 1 µl of secondary antibody in 99 µl of serum at 1% in PBS (dilution of 1 µl of serum in 99 µl of PBS). The secondary antibody is incubated in a humidity chamber for half an hour.

Table S4. Comparison between the biochemical analysis during rehabilitation (ante-mortem) and after euthanasia (post-mortem) with the normal baseline values.

Item	Time	CK (U/L)		cTnI (μg/L)		BUN (mg/dL)		Creatinine (mg/dL)	
		Study animal	Normal value for this species	Study animal	Normal value for this species	Study animal	Normal value for this species	Study animal	Normal value for this species
**Ante-mortem (During rehabilitation period)**	Day 0 (26/04/2019)	837.8	48 to 154 [1]	0.035	Non existing	128	36 to 69 [1]	0.7	1.4 to 2.8 [1]
	Day 1 (27/04/2019)	885.1		0.151		230		0.9	
	Day 2 (28/04/2019)	334.1		0.133		196		0.8	
	Day 3 (29/04/2019)	959.0		0.120		195		0.6	
	Day 4 (30/04/2019)	455.7		0.164		173		0.4	
	Day 5 (01/05/2019)	715.3		0.162		170		0.4	
**Post-mortem (After euthanasia)**	Day 5 (01/05/2019) (1:30 h after euthanasia)	843.6		0.168		171		0.4	

1. Nachtigall, P.E.; Pawloski, J.; Schroeder, J.P.; Sinclair, S. Successful maintenance and research with a formerly stranded Risso's dolphin (Grampus griseus). *Aquat. Mamm.* **1990**, *16*, 8–13.
